# Bioenergetics and Biomechanics of Handcycling at Submaximal Speeds in Athletes with a Spinal Cord Injury

**DOI:** 10.3390/sports8020016

**Published:** 2020-01-29

**Authors:** Gabriela Fischer, Pedro Figueiredo, Luca Paolo Ardigò

**Affiliations:** 1 School of Exercise and Sport Science, Department of Neurosciences, Biomedicine and Movement Sciences, University of Verona, Verona 37131, Italy; luca.ardigo@univr.it; 2 Laboratory of Biomechanics, Department of Physical Education, Federal University of Santa Catarina, Florianópolis 88040-900, Brazil; 3 Portugal Football School, Portuguese Football Federation, Oeiras 1495-433, Portugal; pedfig@me.com; 4 Research Center in Sports Sciences, Health Sciences and Human Development, CIDESD, University Institute of Maia, ISMAI, Maia 4475-690, Portugal

**Keywords:** locomotion, paralympic classification, handbike, paralympic sport, performance

## Abstract

Background: This study aimed at comparing bioenergetics and biomechanical parameters between athletes with tetraplegia and paraplegia riding race handbikes at submaximal speeds in ecological conditions. Methods: Five athletes with tetraplegia (C6-T1, 43 ± 6 yrs, 63 ± 14 kg) and 12 athletes with paraplegia (T4-S5, 44 ± 7 yrs, 72 ± 12 kg) rode their handbikes at submaximal speeds under metabolic measurements. A deceleration method (coasting down) was applied to calculate the rolling resistance and frontal picture of each participant was taken to calculate air resistance. The net overall Mechanical Efficiency (Eff) was calculated by dividing external mechanical work to the corresponding Metabolic Power. Results: Athletes with tetraplegia reached a lower aerobic speed (4.7 ± 0.6 m s^−1^ vs. 7.1 ± 0.9 m s^−1^, P = 0.001) and Mechanical Power (54 ± 15 W vs. 111 ± 25 W, P = 0.001) compared with athletes with paraplegia. The metabolic cost was around 1 J kg^−1^ m^−1^ for both groups. The Eff values (17 ± 2% vs. 19 ± 3%, P = 0.262) suggested that the handbike is an efficient assisted locomotion device. Conclusion: Handbikers with tetraplegia showed lower aerobic performances but a similar metabolic cost compared with handbikers with paraplegia at submaximal speeds in ecological conditions.

## 1. Introduction

The handbike is one of most popular alternative propulsion devices for people with spinal cord injuries and is commonly used in rehabilitation programs [1,2], recreational activities [2,3], and competitions [4,5,6]. Competitive handcycling has existed since the mid-1980s and became an integrated Paralympic Cycling in the Athens 2004 Paralympic Games [2]. In last Rio 2016 Paralympic Games, athletes competed in 13 road race events, an individual time-trial, and a mixed relay [7].

To date, handbikers are grouped in five classes depending on their level of functional ability (from most impaired class—H1—to least impaired—H5) to minimize the impairment impact on competition outcome [8]. Succinctly, athletes who present impaired muscle power, complete trunk stability loss, and limited upper limb function, much like athletes with complete tetraplegia, are classified as H1 and H2. Those presenting poor-to-good trunk control, much like athletes with high and low paraplegia, are included in the H3 and H4 classes. H1–H4 compete using arm powered handbikes, where a recumbent position is mandatory. The H5 class includes those who have excellent trunk balance, like amputee athletes, and mandatorily ride knee seat handbikes [7].

Increasing popularity as Paralympic discipline inspired lighter and more aerodynamic handbikes [9]. Despite its increasing recognition, knowledge about the bioenergetics and biomechanics of athletes riding race handbikes in ecological conditions is scarce. Namely, there is minimal field evaluation in Paralympic sports [10]. Fischer and collaborators [6] were the first to calculate Metabolic Power ($\dot{E}$, J kg^−1^ min^−1^) and metabolic cost (C, J kg^−1^ m^−1^) during a 22-km simulated handcycling time trial in athletes with high paraplegia. According to authors, relatively high speeds (up to 8 m s^−1^) and low C (about 1 J kg^−1^ m^−1^) could be attributed to advances in handbike technology, including the gear system and the reduction of rolling, air resistances, and overall mass.

Rolling and air resistances of seated and knee seat handbikes were quantified by Groen et al. [11] to obtain (external) Mechanical Power ($\dot{W}$*_ext_*). They proposed a power balance model to estimate values of (overall) Mechanical Efficiency (*Eff*, %) in elite handcycling athletes. Mechanical Efficiency quantifies the capability of converting metabolic power into mechanical power, viz. power useful for propulsion [12]. The Mechanical Efficiency in the four athletes tested in Groen et al.’s study [11] was 17.9%. Lovell et al. [13] found similar values (17.2%) testing trained handbikers on an adapted cycle ergometer. Both studies evaluated athletes with low lesion levels and little impaired function. Hence, the economy and *Eff* values of athletes with more severe impairments, such as tetraplegia, are still unknown. Therefore, this study aimed at comparing bioenergetics and biomechanical parameters between athletes with tetraplegia and paraplegia riding race handbikes at submaximal speeds in ecological conditions. We hypothesized that athletes with more severe impairments show lower speeds, economy (i.e., higher C), and *Eff*.

## 2. Materials and Methods

### 2.1. Participants

Seventeen athletes with spinal cord injury participated in this study (5 athletes with tetraplegia and 12 athletes with paraplegia). Specific information on impairment level and duration, sport class classification, and anthropometric characteristics are presented in Table 1. All participants were classified athletes according to the Union Cycliste Internationale (UCI) classification system with at least two years’ experience in handcycling events. This study was approved by the University of Verona Ethics Committee and conformed to standards set by the Declaration of Helsinki. Written informed consent was obtained from all participants.

### 2.2. Handbikes

**Figure 1:**
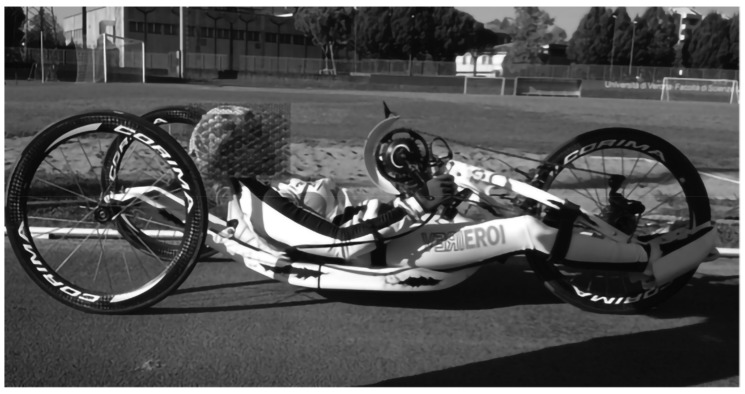
Recumbent handbike.

Each participant used his/her own handbike, with a synchronous crank system, to perform all tests. The majority of the studied handbikes were recumbent models (Figure 1). Tyre pressure was controlled to 8 bars.

### 2.3. Procedures

Handbikers were asked to avoid high-intensity exercise for 48 hours before the test and also to avoid caffeine intake on the morning of the testing day. Participants rode their handbikes on athletics track at submaximal aerobic speeds under cardiorespiratory monitoring. They were equipped with a portable telemetric gas analysis system unit (K4b2, Cosmed, Rome, Italy) for measuring oxygen consumption (VO_2_, mL O_2_ kg^−1^ min^−1^) and respiratory exchange ratio (*RER*) on a breath-by-breath basis. After a 5-min rest, participants started the test at a pre-determined constant “aerobic speed” for at least 4 min to reach a VO_2_ steady state.

To define speeds, we asked participants: “What is your average speed for road races (marathon distance) on flat terrain?” Then, three further speeds, 2, 4, and 6 km h^−1^ slower than average speed, were performed randomly. This procedure allowed guaranteeing aerobic (*RER* < 1) and submaximal speeds to each participant. Gear and arm cranking frequency were freely chosen. Each handbike was equipped with a GNSS/GPS receiver (Edge 305, Garmin, Olathe, KS, USA) allowing participants to control target speed as constant as possible [14]. A rest between trials was administered until oxygen consumption returned to the pre-test value.

### 2.4. Mechanical Tests

A deceleration method (i.e., coasting down) was used on athletics track with tartan surface to calculate rolling resistance. Furthermore, each participant’s frontal picture was taken to calculate air resistance. All tests were performed at same time of day and during an absence of wind (29 ± 4 °C air temperature and 757 ± 4 mmHg atmospheric pressure).

### 2.5. Calculations

#### 2.5.1. Bioenergetics Measurements

Handcycling *C* was obtained from the ratio of Metabolic Power, above resting, to the speed of progression. Metabolic Power was calculated according to *RER* empirical function [15] to obtain VO_2_ metabolic equivalent:

$$\dot{E}={\mathrm{VO}}_{2}\;\left(4.94\times RER+16.04\right)/60$$

#### 2.5.2. Biomechanical Measurements

Level handcycling $\dot{W}$*_ext_* can be divided into two components: the power developed against rolling resistance $\dot{(W_{r}})$ and the power developed against air resistance ($\dot{W_{a}})$. The total resistance ($R_{t}$, N) opposing the handbiker’s level progression is the sum of the rolling resistance ($R_{r}$), due to friction force as the wheels roll along the course, and air resistance ($R_{a}$). Therefore:

$$R_{t}=R_{r}+R_{a}$$

In its turn:

$$R_{r}=M\times C_{r}\times g$$

where *M* is the total athlete + handbike mass (kg), $C_{r}$ is the rolling coefficient, and *g* is acceleration due to gravity (i.e., 9.81 m s^−2^). The power developed against the rolling resistance was obtained multiplying $R_{r}$ by speed.

Coasting down is a simplified deceleration method used to calculate $C_{r}$ [16]. A participant mounted on the handbike just holding the handgrip without hand pedalling, was pushed until he/she reached a speed of about 3 m s^−1^, and then was suddenly left by tester. The decreasing speed was recorded at 1 Hz by means of the GPS receiver. The procedure was performed three times on an athletics track. Decreasing speed data were fitted by a linear regression, where the slope (i.e., the straight line angular coefficient) indicated deceleration due to rolling resistance. The average slope, corresponding to $C_{r}$ × g product, was divided by g to yield $C_{r}$.

$$R_{a}=0.5\times \rho \times A\times C_{a}\times s^{2}$$

where *ρ* is the air density, *A* is the projected frontal area, $C_{a}$ is the aerodynamic coefficient, and *s^2^* is the squared average speed. The projected frontal area was obtained with each participant positioned on his handbike beside a reference (0.8 m height) and 2 m away from a tripod topped by an 8.2 MP digital photography camera (Kodak EASYSHARE C140, Kodak, Rochester, United States) using a photographic method [17]. Frontal pictures were analyzed with Image J (NIH Image 1.62, USA), During bioenergetic measurements, barometric pressure (*P*, mmHg) and air temperature (*T*, K) were recorded with a portable telemetric gas analysis system unit for each test to calculate air density as:

*ρ* = *ρ_°_* (P / 760) × (273 / *T*)

where *ρ_°_* is the density of dry air at a standard temperature and pressure (1.239 m^−3^; [15]).

The aerodynamic coefficient was a constant depending on shape, position, and airflow conditions related, in this case, to the athlete + handbike complex. The aerodynamic coefficient was calculated based on the measurements of one handbiker. The participant had a powermeter (SRM, Jülich, Germany) mounted on his handbike, allowing $\dot{W}$_ext_ to be recorded at vehicle level. The participant rode his handbike at several speeds (from 5.0 to 7.7 m s^−1^) with both a freely chosen arm cranking frequency and fixed arm cranking frequencies (60, 75, 90, and 105 RPM) to check for possible variations in $C_{a}$. Assuming that $\dot{W}$*_ext_* was recorded at vehicle level, i.e., the power measured by means of the powermeter (${\dot{W}}_{extSRM}$) were equivalent to $\dot{W}$*_ext_* as $\dot{W_{r}}$ + $\dot{W_{a}}$, $C_{a}$ could be determined according to Capelli et al.’s approach [12]:

$${\dot{W}}_{extSRM}=\left(0.5\times \rho \times A\times C_{a}\times s^{2}+R_{r}\right)\times s$$

Handcycling *Eff* was calculated at each performed speed as a *ratio* of $\dot{W}$*_ext_* to $\dot{E}$.

### 2.6. Statistical Analysis

Data were checked for normality using the Shapiro–Wilk test and were presented as means ± SD. The group mean differences for participants’ characteristics and all bioenergetics and biomechanical variables were analyzed using independent sample *t* tests. Analysis was performed with IBM SPSS Statistics 20 (IBM Corporation, Armonk, USA). The level of statistical significance was set at P < 0.05. The effect size was computed using Cohen’s d, Hedges’g comparison of groups (with different sample size).

## 3. Results

**Figure 2:**
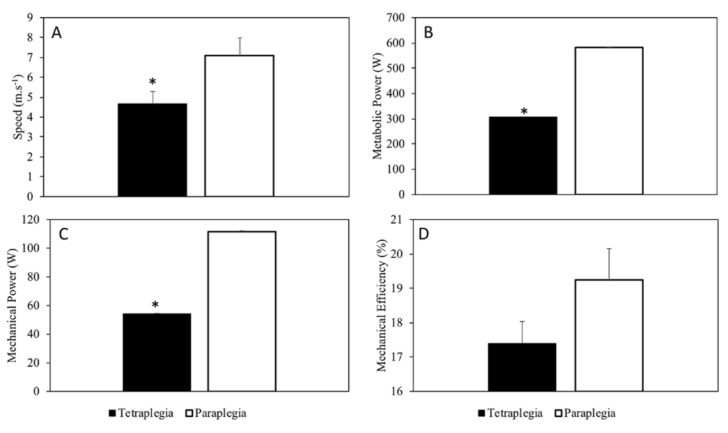
Speed (**A**), Metabolic Power (**B**), (external) Mechanical Power (**C**), and the (overall) Mechanical Efficiency (**D**) of athletes with tetraplegia and paraplegia riding race handbikes.

**Figure 3:**
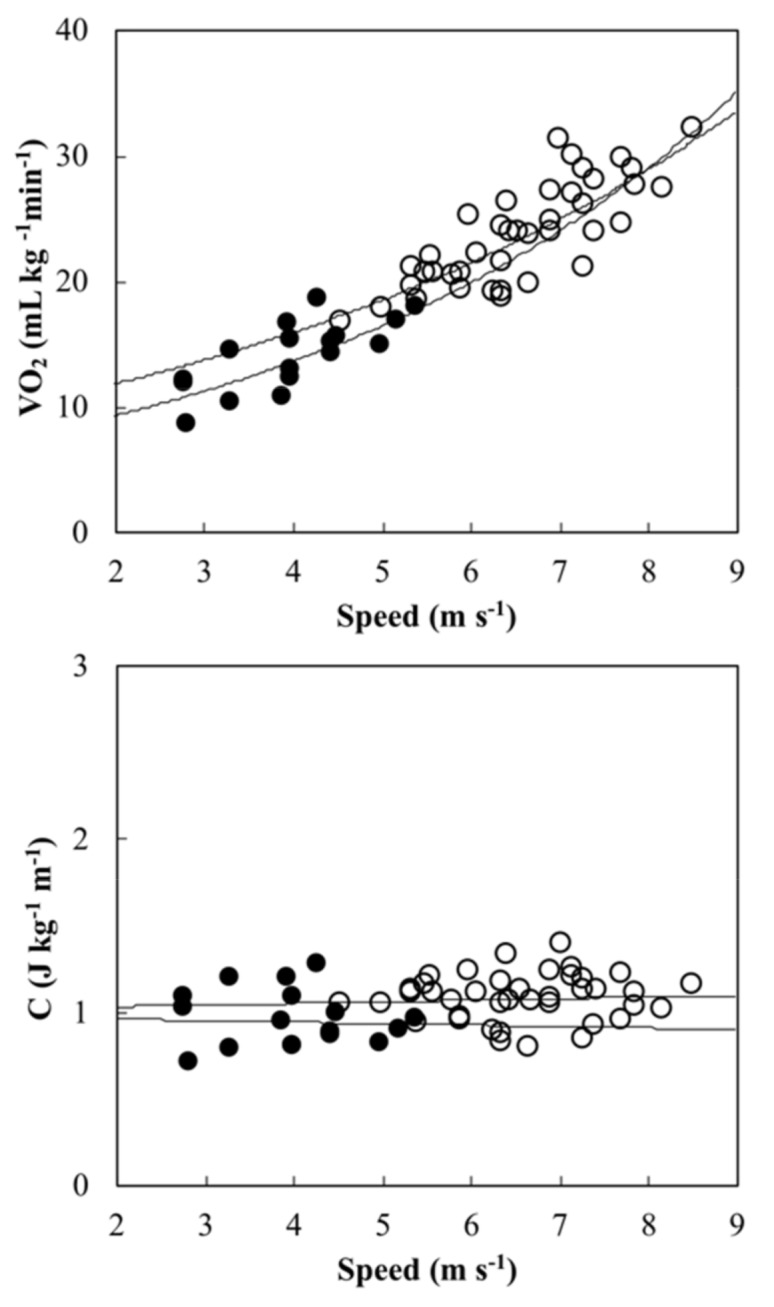
Oxygen consumption (VO_2_) and metabolic cost (C) over speed in athletes with tetraplegia 165 (black circles) and paraplegia (empty circles). Exponential regression lines for graphical purposes only: through values featuring athletes with tetraplegia, VO_2_ = 6.39 e0.189 speed; R² = 0.54; n = 14) and paraplegia 167 (VO_2_ = 8.79 e0.149 speed; R² = 0.64; n = 39).

Age (ES 0.15; small), height (ES 0.17; small), body mass (ES 0.71; moderate), and the duration of injury (ES 0.41; moderate) are shown in Table 1. There was no difference (P > 0.05) in participants’ characteristics between tetraplegia and paraplegia. Despite different handbike adjustments, investigated biomechanical variables (*M*, $C_{r}$, $R_{r}$, and *A*) did not differ between groups (P > 0.05, Table 2). However, a large effect size was found in Mass (1.04) and the rolling coefficient (1.46), while a moderate effect size was found in rolling resistance (0.41) and the project frontal area (0.5). $C_{a}$ resulted 0.71 ± 0.07 with a low coefficient of variation of 9.5% over trials (i.e., over different speeds and different arm cranking frequencies in one participant; Table 2). Figure 2 shows speed (ES 2.89; large), $\dot{E}$, (ES 2.39; large), $\dot{W}$*_ext_* (ES 2.5; large), and *Eff* (ES 0.72; moderate) of athletes with *tetraplegia* and *paraplegia* riding race handbikes. Handbikers with *tetraplegia* showed significantly (P = 0.001) lower values of speed (−34%), $\dot{E\;}$ (−48%), and $\dot{W}$*_ext_* (−52%) compared with handbikers with *paraplegia*. *Eff,* in its turn, did not differ significantly between groups (P = 0.262) despite being lower (−57%) in handbikers with *tetraplegia*. Both groups’ VO_2_ and *C* over speed are shown in Figure 3.

## 4. Discussion

Study compared bioenergetics and biomechanical features between athletes with tetraplegia and paraplegia riding race handbikes at submaximal speeds in ecological conditions. In confirmation of our hypothesis, athletes with paraplegia showed higher speeds but similar economy.

According to Figoni [18], impairments of spinal cord injuries frequently result in two major exercise-related problems: a reduced ability to voluntarily perform large muscle group aerobic exercise and an inability to stimulate the cardiovascular system to support high rates of aerobic metabolism. Our results are in line with such an explanation given that our athletes with tetraplegia rode their handbikes at speeds 34% lower than athletes with paraplegia. Furthermore, differences in $\dot{E}$ and $\dot{W}$*_ext_* could also be explained by less active muscle mass, impaired muscle power, and impaired sympathetic responses in athletes with complete tetraplegia [19]. An interrupted sympathetic response might lead to bradycardia, lower ventilation, insufficient splanchnic blood outflow, and sweating disturbances in tetraplegics compared to paraplegics [20]. On the other hand, individuals with an injury below T5 (i.e., paraplegics) have full supra-spinal sympathetic control of heart and upper body vasculature [21].

Regarding the functional limitations associated with tetraplegia, they may negatively impact on handcycling economy, as well. Namely, we found speeds ranging from 2.8 m s^−1^ to 5.4 m s^−1^ and C of 0.96 ± 0.16 J k^−1^ m^−1^. In case of paraplegia, speed range was higher (4.5 m s^−1^ to 8.50 m s^−1^) causing air resistance to be higher, but C remained around 1 J kg^−1^ m^−1^ (interestingly, still half of its value featuring able-bodied walking, Ardigò et al. [22]). According to Fischer et al. [6], a C value of 1 J kg^−1^ m^−1^ (considering both aerobic and anaerobic contributions) was observed in athletes with paraplegia in a simulated handcycling competition at around 8 m s^−1^. Therefore, our results’ interpretation is that handbikers with tetraplegia rode their handbikes at lower speeds than those with paraplegia but with a very similar amount of C.

Mechanical Efficiency was 17.4 ± 2% with tetraplegia and 19.25 ± 3% with paraplegia, but no statistical differences were observed between them probably due to the limited sample size. Previous studies [3,23,24] performed on attach-unit handbikes, which are usually used for rehabilitation purposes, showed lower Eff values (from 5.6% to 12.2%). In contrast to this, our results agree with values reported in studies performed with a modern handbike ergometer configuration (from 15.8% to 20.5% [25]) and trained handbikers (from 25.3% to 17.2% [13]). Our study is the first to show Eff values considering different injury levels, which are reflected by different sport classes.

“Efficient” locomotion is one where most of metabolic energy input (i.e., $\dot{E}$) is transformed into mechanical energy output (i.e., $\dot{W}$*_ext_*) available for propulsion [12]. For positive work [26], *Eff* cannot exceed 25%–30%. This value can be considered a product of two main components: muscle efficiency and transmission efficiency [27]. Indeed, the handbike device seems to maximize transmission efficiency by means of synchronous propulsion. This could be explained mainly by: i) body weight support by backrest, especially in recumbent models; ii) well-balanced muscle contraction; iii) an optimized force/speed relationship due to wise gears use; iv) an increased base of support and lowered center of mass (providing more stability); v) a reduced rolling resistance due to the use of light-weight materials and the high quality of used hubs; and iv) a drastic reduction of $C_{a}$, especially with recumbent models.

We did not find any significant between-group differences in handbike biomechanical variables (Table 2). Groen et al. [11] reported a lower $C_{r}$ value (0.0039) while testing a racing handbike (Schmicking, Holzwickede, Germany) on a synthetic smooth surface, which could explain such a finding. $C_{r}$ was also calculated for attach-unit handbike models showing values of 0.0131 [24] and 0.00794 [3]. However, $C_{r}$ different values should be compared to each other with caution, because they depend very much on factors such as surface’s type, inflation pressure, the number of wheels [28], the diameter of the wheels, and the tyre manufacture [29]. The projected frontal area was comparable to that measured with some racing bicycles in a dropped posture [30], i.e., 0.34 m^2^ and a recumbent bike (Easy Racer, [29]), i.e., 0.35 m^2^. Furthermore, since 2009 International Paralymic Committee has allowed back support angles lower than 45°, meaning that athletes are able to adopt more lying positions. Nevertheless, athlete must still have—by UCI regulation—a clear sight, i.e., the horizontal of his eye-line must be above the crank set [7]. Complying with such a rule, most aerodynamic athlete/handbike evaluated in this study showed an *A* value of 0.32 m^2^. Consequently, $C_{a}$ (0.71) was similar to those calculated for two-wheeled recumbent bikes (0.77, [29]). Groen et al. [11] reported values of $C_{a}$ of 0.83 and *A* of 0.40 m^2^ while testing a racing handbike model.

Some of the present study’s limitations need to be addressed. The fact that the study was performed following an ecological approach made controlling the sample for homogeneity (i.e., participants’ training status, handbikes, and disability-causing injuries) very difficult. The study tried to keep competitive handcycling in the ecological setting as much as possible. However, protocol was performed on an unusual (for handbikers) athletics track. The tartan surface offered higher-than-usual friction resistance, as reported by participants. High friction resistance could have biased investigate performances. Additionally, the air resistance was calculated for each subject by using $C_{a}$ measured on only one subject riding a recumbent handbike. We believe that the above limitations do not excessively weaken the relevance of the study, which provided, what we consider to be, important insights about submaximal performance in athletes with tetraplegia and paraplegia during handcycling.

## 5. Conclusions

In conclusion, handbikers with tetraplegia showed lower aerobic performances but similar metabolic costs compared with handbikers with paraplegia at submaximal speeds in ecological conditions. A similar mechanical efficiency for both groups suggested that the handbike is an efficient assisted locomotion device irrespective of the level of spinal cord injury.

## Figures and Tables

**Table 1:** Subjects’ anthropometric characteristics, impairment features, and sport classes.

	Age (yrs)	Height (cm)	Body Mass (kg)	Injury Description	Duration of Injury (yrs)	Sport Class
Tetraplegia						
1	37	185	77	C6/7 incomplete	9	MH1
2	42	170	44	C6/7 incomplete	9	MH1
3	50	171	73	C6/7 complete	17	MH1
4	36	178	73	C8/T1 incomplete	16	MH2
5	50	160	49	C6/7 complete	21	WH1
Mean (SD)	43(6)	173(8)	63(14)		14(5)	
Paraplegia						
1	54	173	60	T6/8 complete	34	MH3
2	42	175	75	T4/5 complete	9	MH3
3	44	174	65	T6/7 complete	15	MH3
4	37	174	64	T5 complete	11	MH3
5	43	168	57	T6/7 complete	15	MH3
6	33	170	62	T5/9 incomplete	13	WH3
7	48	183	95	S1-S5 incomplete	Birth	MH4
8	41	182	80	T12 incomplete	8	MH4
9	48	170	74	L1/3 incomplete	24	MH4
10	42	175	87	T8 incomplete	22	MH4
11	38	170	65	S1-S5 incomplete	12	MH4
12	55	168	74	L1 incomplete	20	MH4
Mean (SD)	44(7)	174(5)	72(12)		17(8)	

(MH – men athletes; WH – Women athletes)

**Table 2:** Total mass (M) (athlete plus handbike), the rolling coefficient on the tartan surface (*Cr*), the rolling resistance on the tartan surface (*Rr*), air density (*ρ*), and the projected frontal area (*A*) of athletes and their handbikes.

	*M* (kg)	*C_r_*	*R_r_* (N)	*A* (m^2^)	*ρ* (kg m^−3^)	*C_a_*
Tetraplegia					
1	19	0.0097	9.13	0.36	1.15
2	17	0.0103	9.16	0.33	1.17
3	22	0.0075	6.99	0.49	1.19
4	17	0.0105	9.06	0.38	1.15
5	15	0.0095	5.77	0.33	1.14
Mean(SD)	18(2.4)	0.0095(0.0011)	8.02(1.40)	0.38(0.06)	1.16 (0.02)
Paraplegia					
1	17	0.0074	5.56	0.40	1.15
2	19	0.0080	7.41	0.37	1.15
3	15	0.0086	6.77	0.37	1.19
4	15	0.0078	6.06	0.30	1.16
5	12	0.0085	5.78	0.30	1.14
6	14	0.0067	4.99	0.33	1.19	0.71
7	17	0.0086	9.48	0.53	1.17
8	16	0.0084	7.95	0.32	1.16
9	16	0.0107	9.44	0.32	1.15
10	16	0.0106	10.76	0.33	1.16
11	14	0.0074	5.75	0.32	1.20
12	19	0.0084	7.68	0.34	1.14
Mean(SD)	16(2.0)	0.008(0.001)	7.30(1.83)	0.35(0.06)	1.16(0.02)
